# Highly Pathogenic Avian Influenza A(H5N1) Virus in Wild Red Foxes, the Netherlands, 2021

**DOI:** 10.3201/eid2711.211281

**Published:** 2021-11

**Authors:** Jolianne M. Rijks, Hanna Hesselink, Pim Lollinga, Renee Wesselman, Pier Prins, Eefke Weesendorp, Marc Engelsma, Rene Heutink, Frank Harders, Marja Kik, Harry Rozendaal, Hans van den Kerkhof, Nancy Beerens

**Affiliations:** Dutch Wildlife Health Centre, Utrecht University, Utrecht, the Netherlands (J.M. Rijks, H. Hesselink, M. Kik);; Stichting Faunavisie Wildlife Care, Westernieland, the Netherlands (P. Lollinga);; Stichting Faunavisie Wildlife Care, Blijham, the Netherlands (R. Wesselman);; Dierenkliniek Winsum, Winsum, the Netherlands (P. Prins);; Wageningen Bioveterinary Research, Lelystad, the Netherlands (E. Weesendorp, M. Engelsma, R. Heutink, F. Harders, N. Beerens);; Dutch Food and Consumer Products Safety Authority, Utrecht (H. Rozendaal);; Coordination Centre for Communicable Disease Control, National Institute for Public Health and the Environment, Bilthoven, the Netherlands (H. van den Kerkhof)

**Keywords:** influenza A virus, H5N1 subtype, foxes, animals, wild, influenza in birds, amino acids, disease outbreaks, Vulpes vulpes, viruses, respiratory infections, zoonoses, the Netherlands

## Abstract

We detected infection with highly pathogenic avian influenza A(H5N1) virus clade 2.3.4.4b in 2 red fox (*Vulpes vulpes*) cubs found in the wild with neurologic signs in the Netherlands. The virus is related to avian influenza viruses found in wild birds in the same area.

On May 10, a red fox *Vulpes vulpes* cub (cub 1) displaying abnormal behavior was found in Bellingwolde, the Netherlands, and taken into care of a wildlife rescue center. Upon entry, the 6- to 8-week-old cub was slightly dehydrated and showed at intervals of <30 minutes lip retraction, rapid opening and closing of mouth, excessive salivation, skin twitching, head shaking, and body tremors (Figure; Video). The cub first seemed to improve, but on May 12 it reacted aggressively when touched. Subsequently, we observed difficult swallowing and labored breathing. The cub seemed blind and stopped eating. As the situation further deteriorated, we humanely euthanized the cub on May 16. On May 13, the center received another 6- to 8-week-old red fox cub (cub 2) found ≈900 m from cub 1. Cub 2 was hypothermic and dehydrated. It had seizures and died overnight.

**Figure:**
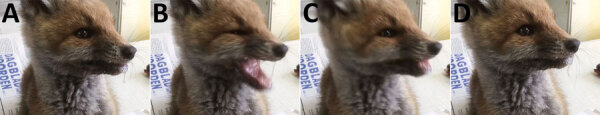
Salivating red fox (*Vulpes vulpes*) cub 1 during a fit, the Netherlands, 2021. Seizure started with retracting lips at 0 sec (A), followed by facial wrinkling with opening of mouth at 0.07 sec (B), closing of the jaws at 0.17 sec (C), then back to “normal” at 0.40 sec (D), before this sequence starts all over at 0.50 sec.

**Video:** Salivating red fox (*Vulpes vulpes*) cub 1 during a fit, the Netherlands, May 10, 2021.

Retrospectively, we concluded that the mother of the cubs was likely a vixen found walking circles on May 10, ≈975 m direct distance from cub 1 and ≈90 m from cub 2. The vixen reacted very aggressively to capture, responding to sound but blind. The vixen had a fresh elbow fracture, probably caused by a road traffic accident. We humanely euthanized her the same day and sent her carcass for destruction.

Although rabies lyssavirus is unlikely in the Netherlands, European bat 1 lyssavirus is endemic in serotine bats (*Eptesicus serotinus*) (*1*). To exclude lyssavirus infection in the fox cubs, we performed a direct fluorescent antibody test on smears of brain tissue in accordance with World Organisation for Animal Health (OIE) protocol (https://www.oie.int/fileadmin/Home/eng/Health_standards/tahm/3.01.17_RABIES.pdf). Test results were negative.

Subsequently, we tested brain samples for avian influenza virus by using a PCR detecting the influenza A virus matrix gene, followed by the subtype-specific H5-PCR on the hemagglutinin gene, as described previously (*2*). The samples from both cubs tested positive (Table), and we subtyped the virus as highly pathogenic avian influenza (HPAI) influenza virus A subtype H5N1. We isolated the HPAI H5N1 virus from the brain of cub 1 by inoculation of the samples into 10-day-old embryonated special pathogen–free chicken eggs.

**Table:** Results of diagnostic tests of avian influenza viruses detected in 2 red fox (*Vulpes vulpes*) cubs, the Netherlands, 2021*

Red fox	Pooled samples of brain	M-PCR1 C_t_ value	M-PCR2 C_t_ value	H5-PCR C_t_ value
Cub 1	Ammon’s horn and medulla oblongata	21.27	21.46	23.35
Cub 1	Cerebellum and cerebrum	19.31	20.79	21.65
Cub 2	Ammon’s horn and medulla oblongata	25.72	25.92	26.44
Cub 2	Cerebellum and cerebrum	20.09	21.58	23.47

*C_t_, cycle threshold; H5-PCR, subtype-specific PCR on the H5 gene; M-PCR1, PCR on the matrix gene of influenza A, repetition 1; M-PCR2, PCR on the matrix gene of influenza A, repetition 2

During April–May 2021, large numbers of dead barnacle geese (*Branta leucopsis*) were reported in the northern part of the Netherlands, and later other species of waterfowl and birds of prey were also found dead. A selected number of dead wild birds were submitted for AI diagnostics and tested positive for HPAI H5N1 virus. We performed whole-genome sequencing of the HPAI H5N1 viruses found in wild birds and the 2 foxes as previously described (*3*) and conducted genetic and phylogenetic analyses to study the relationship between these viruses. Phylogenetic analysis of the gene segments (Appendix 1 Figure 1–8) showed the viruses detected in wild birds and the 2 foxes were in the same cluster and highly related. We classified the viruses as H5 clade 2.3.4.4b viruses, which were related to other HPAI H5N1 viruses detected in wild birds and poultry in Europe during 2020–2021. The HPAI H5N1 viruses detected in the foxes were not related to zoonotic H5N1 strains infecting humans in Asia and did not contain any known zoonotic mutations (data not shown). The sequences of the viruses detected in cub 1 (GISAID [https://www.gisaid.org] accession no. EPI_2194218) and cub 2 (GISAID accession no. EPI_2194219) were identical; the closest related virus was identified in a white-tailed eagle (*Haliaeetus albicilla*) near the village of Noordlaren. We observed only 6 aa differences: mutations A152T and T521I in polymerase basic protein 2 (PB2); M644V in polymerase basic protein 1; A336T in nucleoprotein; L22S in neuraminidase protein; and D209N in nonstructural protein (Appendix 1 Figure 1–8). Whether these changes are associated with adaptation of the avian virus to mammal species remains unknown.

These 2 cases of infection with H5N1 clade 2.3.4.4b virus in wild red fox cubs underscore the need to raise awareness that HPAI viruses are not only zoonotic but also infect other mammal species. HPAI infection should be on the list of differential diagnoses for animals that have signs of respiratory or neurologic disease. The detection of virus in the brain suggests systemic infection of the cubs. The clinical signs were largely consistent with those reported in other natural infections of carnivores with HPAI H5 subtypes (*4*–*7*). Whether the fox cubs were infected through the parents or by eating infected bird carcasses is unclear (cubs start eating solid food at 4 weeks of age). Carnivores are known to be at risk for avian influenza virus infection upon ingesting infected birds (*4*,*5*,*8*). We did not test for virus shedding in these cubs, but virus shedding has been observed in experimental infection of 6- to 10-month-old red foxes with HPAI H5N1 clade 2.2 virus (*8*).

The United Kingdom reported infection of a red fox and seals in an animal shelter with a related HPAI H5N8 clade 2.3.4.4b virus (*9*), and Russia reported infection in poultry workers (*10*). These findings suggest that HPAI H5 clade 2.3.4.4b viruses may sporadically transmit from birds to mammals, including humans. Virus evolution and adaptive mutations must be closely monitored to rapidly identify viruses with increased zoonotic potential.

Appendix 1 Additional information about highly pathogenic avian influenza A(H5N1) virus in wild red foxes, the Netherlands, 2021.

Appendix 2 Acknowledgment of authors and submitting laboratories of the sequences from the GISAID EpiFlu Database used in a study of highly pathogenic avian influenza A(H5N1) virus in wild red foxes, the Netherlands, 2021.
