# Supplementary figures and images for: Highly Pathogenic Avian Influenza A(H5N1) Virus in Wild Red Foxes, the Netherlands, 2021
Source: Emerg Infect Dis. 2021 Nov;27(11):2960–2. doi: 10.3201/eid2711.211281 (PMC8544991; doi:10.3201/eid2711.211281)

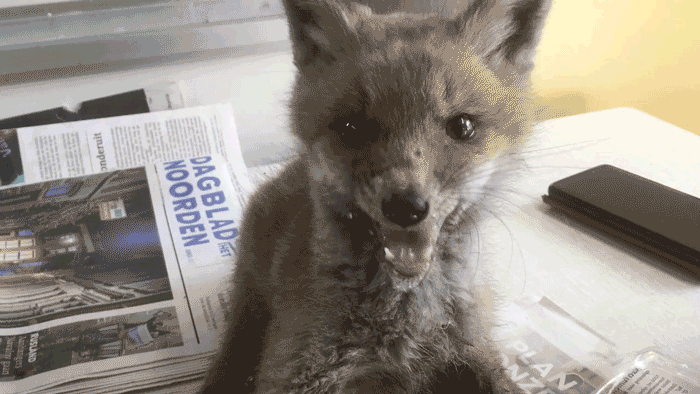

Supplement: Supplementary file 1 [file 21-1281-V.gif]
