# Supplementary material for: Highly Pathogenic Avian Influenza A(H5N1) Virus in Wild Red Foxes, the Netherlands, 2021
Source: Emerg Infect Dis. 2021 Nov;27(11):2960–2. doi: 10.3201/eid2711.211281 (PMC8544991; doi:10.3201/eid2711.211281)
Supplement: Appendix 1 — Additional information about highly pathogenic avian influenza A(H5N1) virus in wild red foxes, the Netherlands, 2021. [file 21-1281-Techapp-s1.pdf]

Article DOI: https://doi.org/10.3201/eid2711.211281

Highly Pathogenic Avian Influenza A(H5N1) Virus in Wild Red Foxes, the Netherlands, 2021

Appendix 1

Methods

Phylogenetic analysis was performed for each gene segment as previously described (*1*). Selected related sequences were obtained from GISAID's EpiFlu database on May 26, 2021 (http://www.gisaid.org) (*2*). Sequences were aligned using MAFFT v7.427 (*3*). Maximum likelihood trees based on the general time reversible model with a gamma-distributed variation of rates and 1000 bootstraps were generated using RAxML v8.2.12 (*4*) and visualized using FigTree 1.4.4 (https://github.com/rambaut/figtree/releases). GISAID accession numbers of the sequences and bootstrap values above 50 are shown in the phylogenetic trees. H5N1 virus sequences originating from the fox cubs are marked in red, high pathogenic avian H5N1 virus sequences from the Netherlands (2020–2021) are marked in green.

References

1. Beerens N, Heutink R, Harders F, Roose M, Pritz-Verschuren SBE, Germeraad EA, et al. Incursion of Novel Highly Pathogenic Avian Influenza A(H5N8) Virus, the Netherlands, October 2020. Emerg Infect Dis. 2021;27:1750–3. PubMed https://doi.org/10.3201/eid2706.204464

2. Shu Y, McCauley J. GISAID: Global initiative on sharing all influenza data - from vision to reality. Euro Surveill. 2017;22:30494. PubMed https://doi.org/10.2807/1560-7917.ES.2017.22.13.30494

3. Katoh K, Standley DM. MAFFT multiple sequence alignment software version 7: improvements in performance and usability. Mol Biol Evol. 2013;30:772–80. PubMed https://doi.org/10.1093/molbev/mst010

4. Stamatakis A. RAxML version 8: a tool for phylogenetic analysis and post-analysis of large phylogenies. Bioinformatics. 2014;30:1312–3. PubMed https://doi.org/10.1093/bioinformatics/btu033

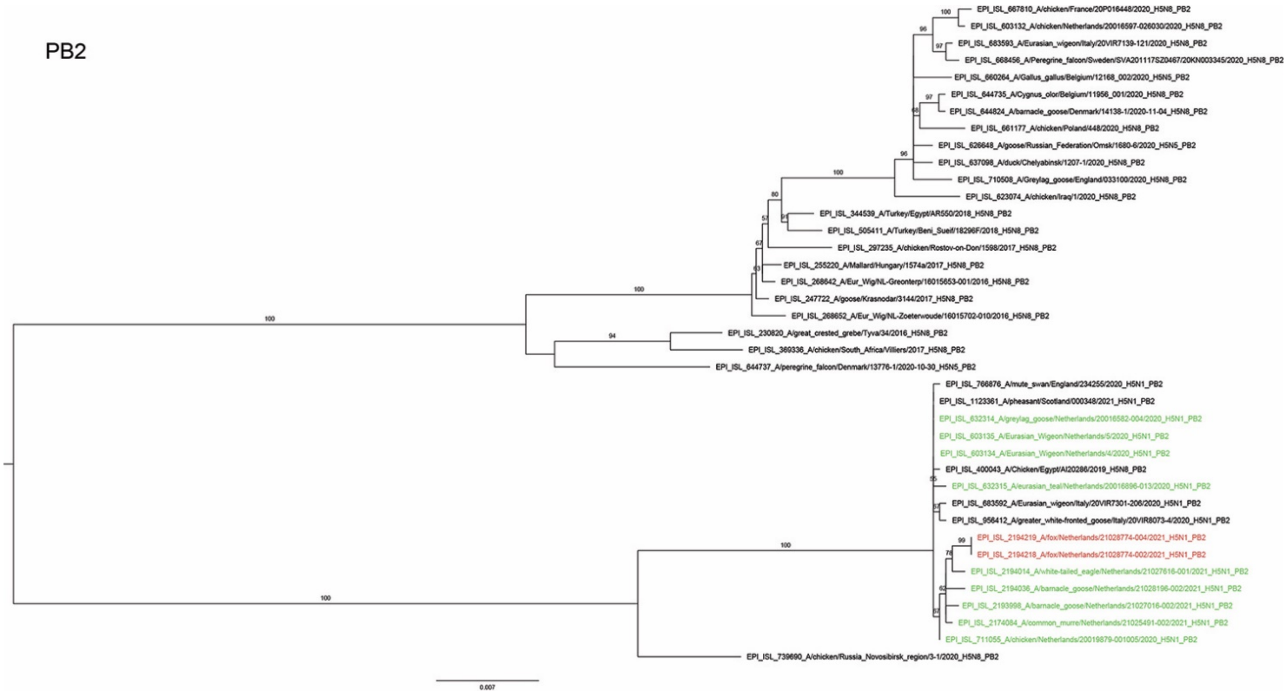

Appendix 1 Figure 1. Phylogenetic tree of PB2 gene segment.

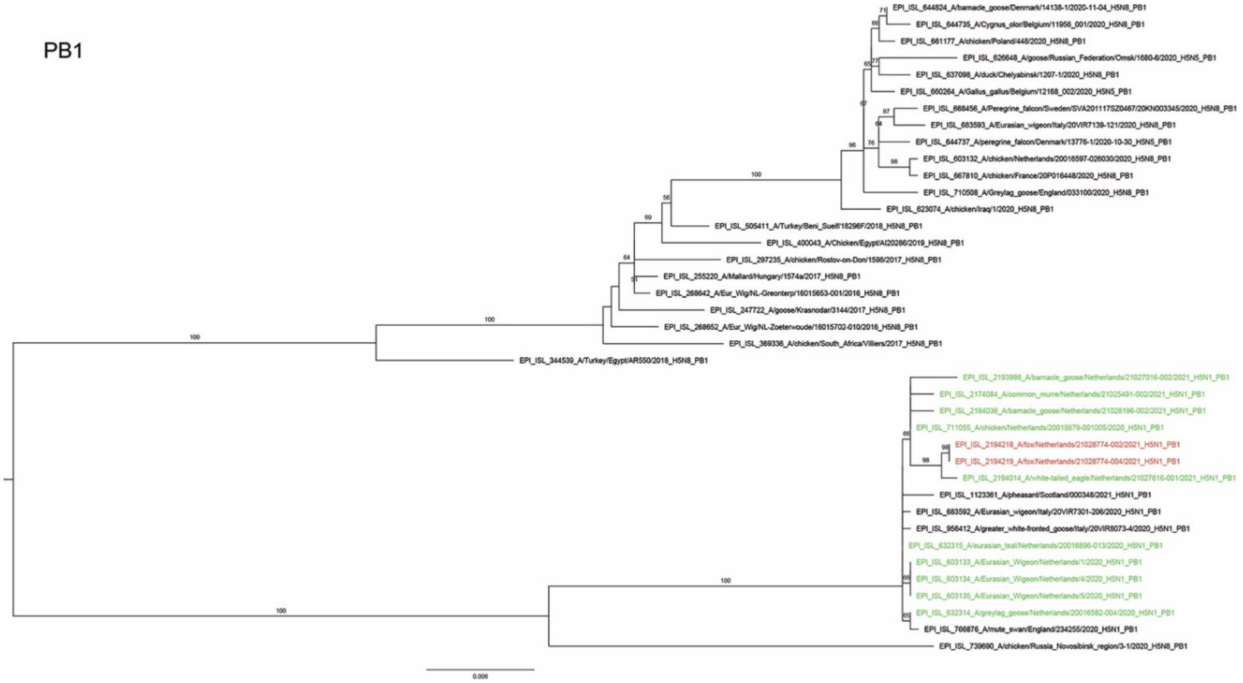

Appendix 1 Figure 2. Phylogenetic tree of PB1 gene segment.

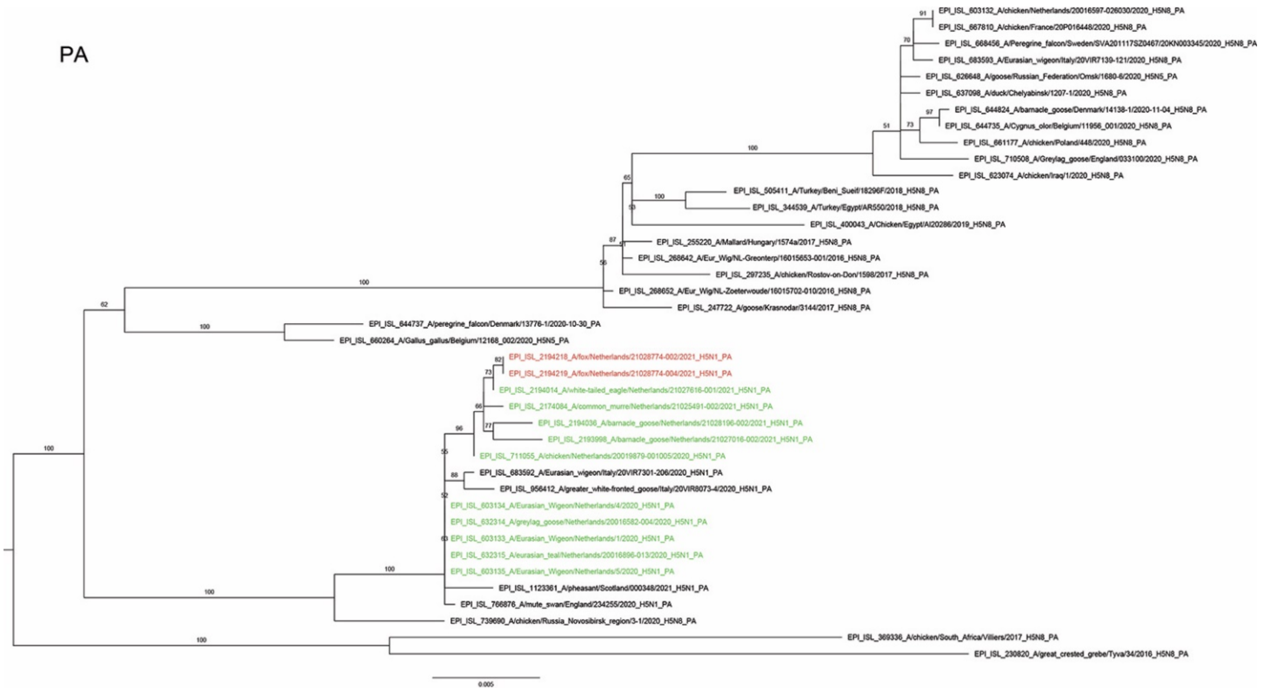

Appendix 1 Figure 3. Phylogenetic tree of PA gene segment.

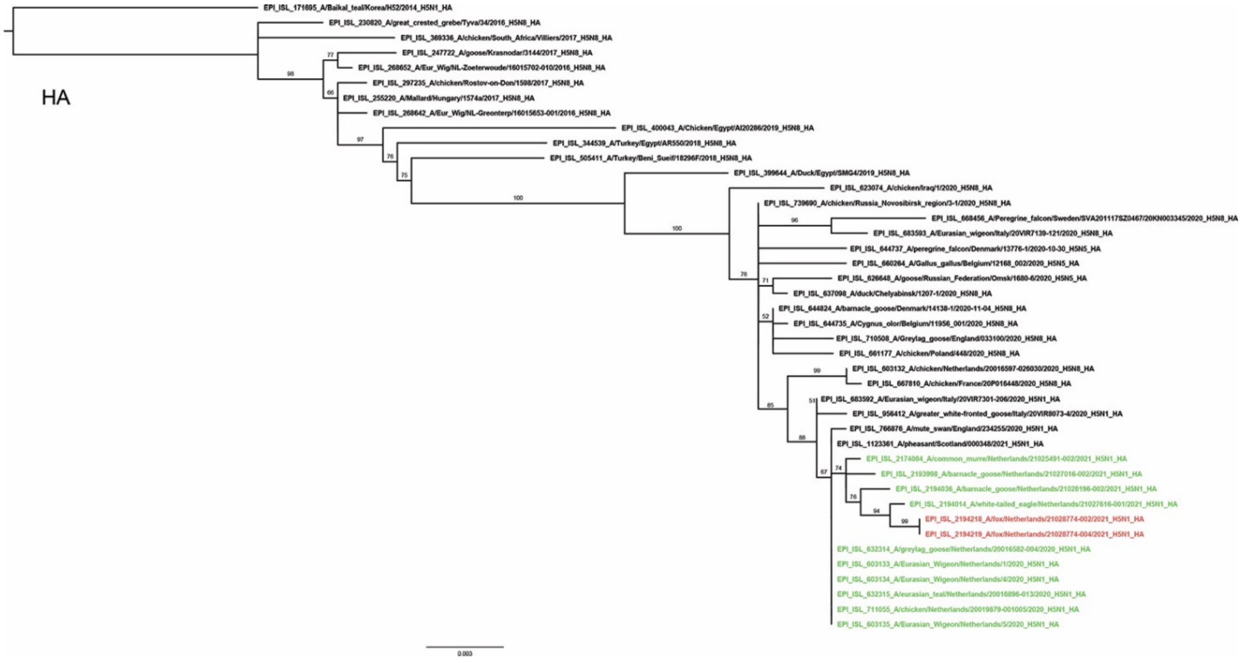

Appendix 1 Figure 4. Phylogenetic tree of HA gene segment.

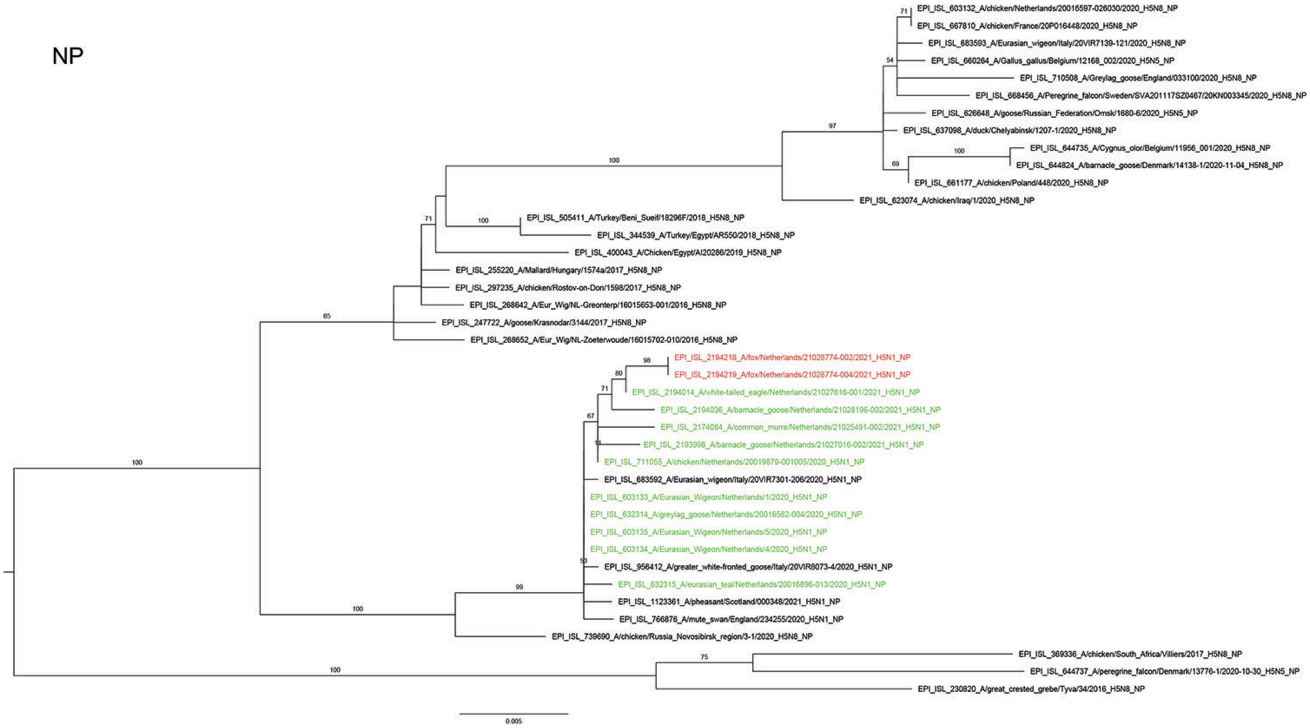

Appendix 1 Figure 5. Phylogenetic tree of NP gene segment.

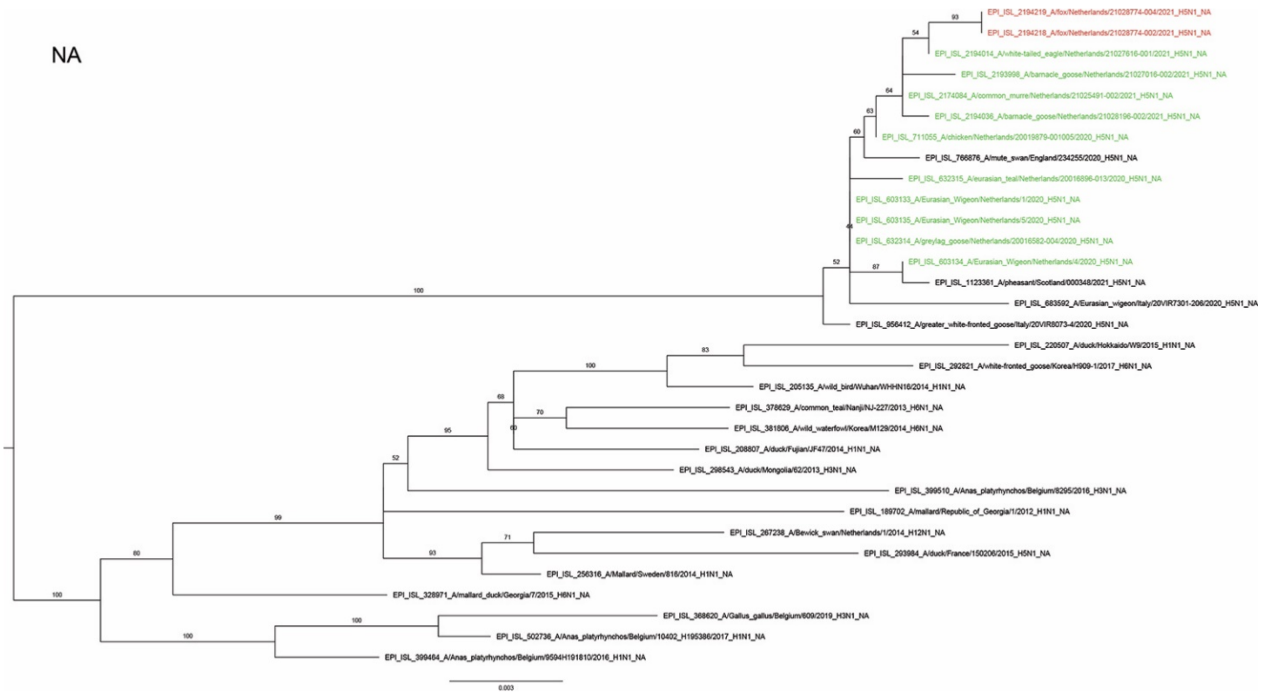

Appendix 1 Figure 6. Phylogenetic tree of NA gene segment.

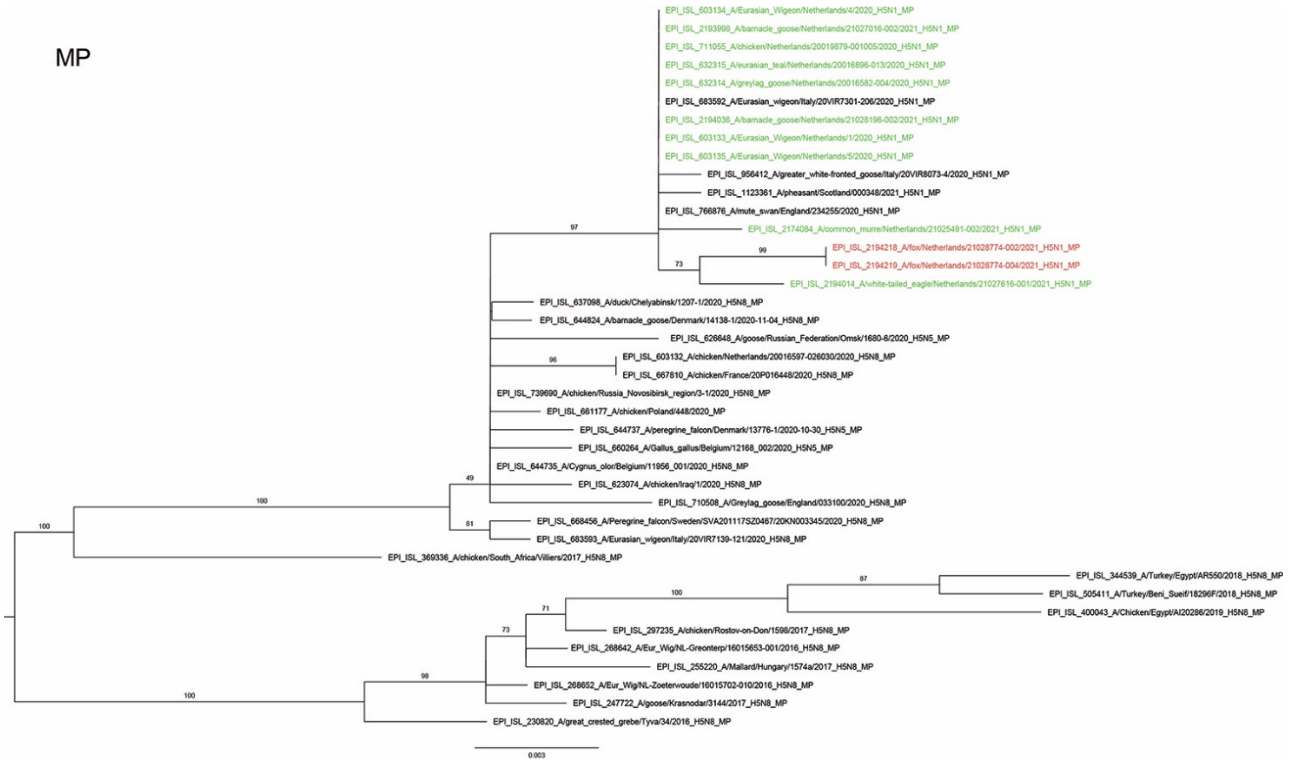

Appendix 1 Figure 7. Phylogenetic tree of MP gene segment.

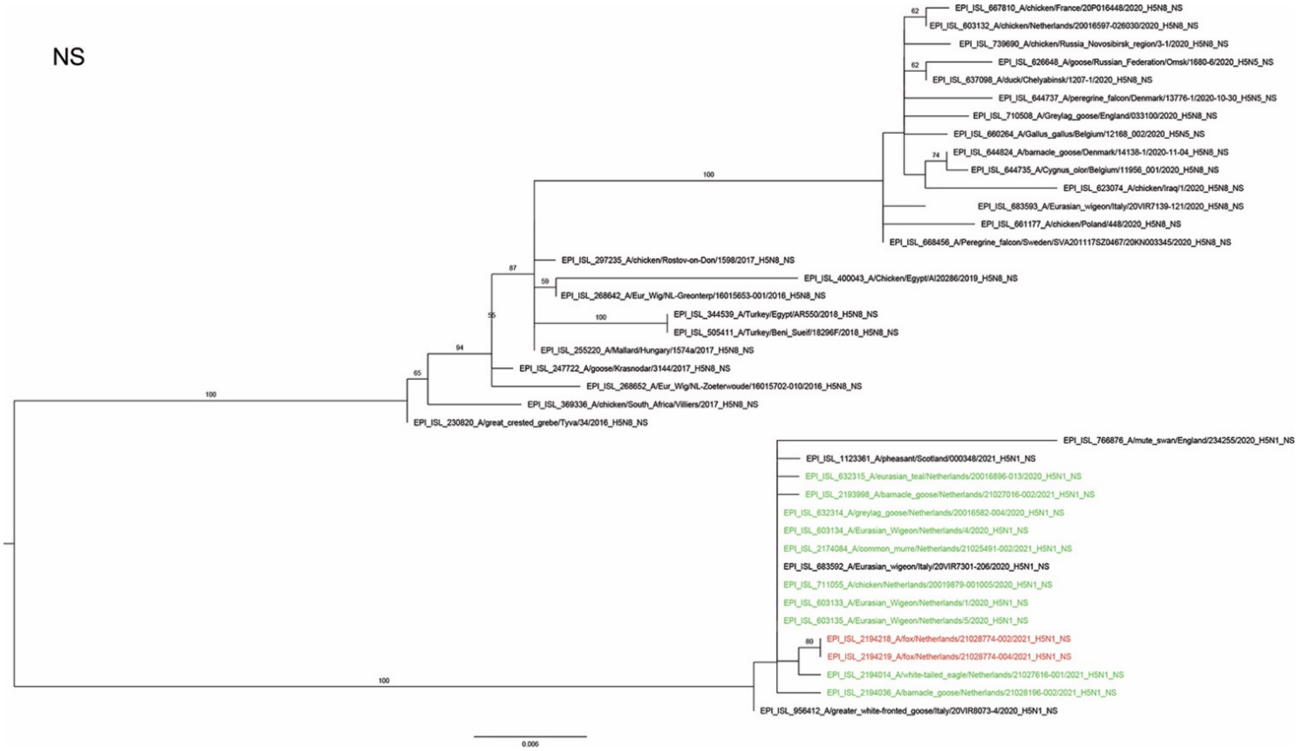

Appendix 1 Figure 8. Phylogenetic tree of NS gene segment.
